# Inhibition of Cyclin-Dependent Kinase 9 Downregulates Cytokine Production Without Detrimentally Affecting Human Monocyte-Derived Macrophage Viability

**DOI:** 10.3389/fcell.2022.905315

**Published:** 2022-05-26

**Authors:** Brian J. McHugh, Jillian Stephen, Calum T. Robb, Sarah Fox, Tiina Kipari, Jennifer A. Cartwright, Christopher Haslett, Rodger Duffin, Christopher D. Lucas, Adriano G. Rossi

**Affiliations:** University of Edinburgh Centre for Inflammation Research, Queen’s Medical Research Institute, Edinburgh BioQuarter, Edinburgh, United Kingdom

**Keywords:** macrophage, cyclin-dependent kinase inhibitor, resolution of inflammation, efferocytosis, cytokine production

## Abstract

Cyclin-dependent kinase (CDK) inhibitor drugs (CDKi), such as R-roscovitine and AT7519, induce neutrophil apoptosis *in vitro* and enhance the resolution of inflammation in a number of *in vivo* models. This class of compounds are potential novel therapeutic agents that could promote the resolution of acute and chronic inflammatory conditions where neutrophil activation contributes to tissue damage and aberrant tissue repair. In this study we investigated CDKi effects on macrophage pro-inflammatory mediator production and viability. Treatment of human monocyte-derived macrophages (MDMs) with the CDKi AT7519 and R-roscovitine at concentrations that induce neutrophil apoptosis had no significant effect on control or LPS-activated MDM apoptosis and viability, and did not detrimentally affect MDM efferocytosis of apoptotic cells. In addition, enhanced efferocytosis, induced by the glucocorticoid dexamethasone, was also unaffected after a short time treatment with R-roscovitine. Macrophage cytokine responses to inflammatory stimuli are also of importance during inflammation and resolution. As a key target of CDKi, CDK9, is involved in protein transcription via the RNA polymerase II complex, we investigated the effect of CDKi drugs on cytokine production. Our data show that treatment with AT7519 significantly downregulated expression and release of key MDM cytokines IL-6, TNF, IL-10 and IL-1β, as well as markers of pro-inflammatory macrophage polarisation. R-Roscovitine was also able to downregulate inflammatory cytokine protein secretion from MDMs. Using siRNA transfection, we demonstrate that genetic knock-down of CDK9 replicates these findings, reducing expression and release of pro-inflammatory cytokines. Furthermore, overexpression of CDK9 in THP-1 cells can promote a pro-inflammatory phenotype in these cells, suggesting that CDK9 plays an important role in the inflammatory phenotype of macrophages. Overall, this study demonstrates that pharmacological and genetic targeting of CDK9 inhibits an inflammatory phenotype in human MDMs. As such these data indicate that CDK9 may be key to therapeutically targeting pro-inflammatory macrophage functions during chronic inflammation.

## Introduction

Inflammation is the body’s, usually protective, natural response to injury or infection and is driven by innate effector cells such as neutrophils and macrophages (Medzhitov, 2010). A key event terminating the inflammatory response is the resolution of inflammation with subsequent return to tissue homeostasis (Buckley et al., 2013; Headland and Norling, 2015). Inflammation resolution is an active process involving a number of highly regulated events (Nathan, 2002). Two key processes are 1) apoptosis of granulocytes such as neutrophils (Fox et al., 2010) and 2) efferocytosis (Savill et al., 1989; Bratton and Henson, 2011) (phagocytosis and clearance of apoptotic cells by phagocytes such as macrophages). If there is dysregulation in either of these events, neutrophils persist at the site of inflammation, possibly by delayed apoptosis, where they can become inappropriately activated, releasing toxic tissue damaging intracellular granule products and reactive oxygen radicals. Apoptotic cells if not cleared efficiently can also undergo “secondary” necrosis, again releasing their harmful contents (Poon et al., 2014). These events cause tissue damage and amplification of the inflammatory response implicated in the pathogenesis of many inflammatory diseases (Michlewska et al., 2007) such as type 1 diabetes (Clark et al., 2017), atherosclerosis (Viola and Soehnlein, 2015) and chronic pulmonary obstructive disorder (COPD) (Rovina et al., 2013). Many of the current therapies used to treat chronic inflammatory diseases target pro-inflammatory mediator synthesis or function. Alternatively, we have focussed on targeting the resolution phase of inflammation through the selective promotion of granulocyte apoptosis and/or enhancement of efferocytosis (Cartwright et al., 2019). Specifically, we are interested in targeting cyclin-dependent kinases (CDKs), a group of kinases identified for their roles in regulating cell cycle, gene transcription and mRNA processing. We previously demonstrated that pharmacological inhibition of CDKs with R-roscovitine, DRB (5,6-Dichloro-1-beta-D-ribofuranosylbenzimidazole) and AT7519 induces neutrophil apoptosis and promotes the resolution of inflammation in different inflammatory models, suggesting these inhibitors may have therapeutic potential (Rossi et al., 2006; Lucas et al., 2014). Further, our work showed that CDK inhibitor drugs (CDKi) induced neutrophil apoptosis by targeting CDK9 and/or CDK7 (Leitch et al., 2012). CDK9, a serine threonine protein kinase, partners with its regulatory subunit CyclinT1 to form the positive transcription factor elongation complex b (P-TEFb) which acts to phosphorylate RNA polymerase II and promote transcription, particularly of short-lived proteins that promote cell survival - this complex is a key regulator of transcription of the anti-apoptotic protein Mcl-1 and inflammatory cytokines such as TNF (MacCallum et al., 2005). Targetting CDK9 with CDKi prevents its ability to induce gene transcription via phosphorylation of RNA polymerase II and formation of P-TEFb (Rossi et al., 2006; Leitch et al., 2012), thus leading to Mcl-1 degradation and subsequent neutrophil apoptosis.

It is important when targeting increased apoptotic cell burden as a therapeutic option to ensure that these potentially toxic cells are cleared efficiently and “silently” by phagocytes such as macrophages. As such, we investigated CDKi drugs directly on inflammatory macrophages, with the overall aim of determining whether these compounds represent a novel therapeutic strategy to promote the resolution of inflammation in inflammatory diseases. We focused on AT7519 as one of a generation of CDKi molecules with an increased specificity for CDK9 (IC_50_ of <10 nM) (Wyatt et al., 2008; Squires et al., 2009) (Bach et al., 2005), in contrast to other CDKi drugs such as R-roscovitine and DRB which are less potent (IC_50_ of <1 µM and 3 µM respectively) and target a broader range of CDKs (CDK2, 7 and 9 for R-roscovitine and CDK7, 8 and 9 for DRB). A comprehensive comparison of CDKi drugs, including chemical structures, is discussed in our review (Cartwright et al., 2019). We show that unlike treatment of neutrophils, AT7519 at the concentration and time points tested does not induce the apoptosis of human monocyte-derived macrophages (MDMs) and downregulates inflammatory cytokine secretion and markers of an inflammatory phenotype. Furthermore, short term AT7519 treatment of MDMs does not affect the ability of these cells to clear apoptotic neutrophils via efferocytosis. Importantly, we also used siRNA-mediated knockdown of CDK9 in MDMs and performed overexpression of CDK9 in THP-1 cells to specifically investigate CDK9 in macrophage function. This study demonstrates that pharmacological and genetic targeting of CDK9 inhibits polarisation towards a pro-inflammatory phenotype in human MDMs without significantly affecting their initial ability to undergo efferocytosis, a key step in the resolution of inflammation. As such these data indicate that CDK9 inhibitor drugs may represent key therapeutic agents for the treatment of inflammatory diseases especially where neutrophils and macrophages have been implicated. Indeed, given the role of these innate immune cells in diseases such as ARDS, and findings using CDKi in models of acute lung injury we postulate that CDKi may represent a novel therapeutic strategy for the treatment of such diseases. In addition, given findings that neutrophils and especially macrophages are implicated in the pathogenesis COVID-19 (Dorward et al., 2021) such drugs may be useful in treatment of this disease.

## Materials and Methods

### Cell Culture

For leukocytes isolated from healthy volunteer blood, informed written consent was obtained from all subjects. Ethics approval was obtained from the local Lothian Research Ethics Committee (AMREC 15-HV-013 and CIR 20-HV-069). Human MDMs were prepared from peripheral blood as described by our group (Ward et al., 1999). Briefly, peripheral blood mononuclear cells and granulocytes were isolated from whole blood using isotonic discontinuous Percoll^®^ gradients. CD14^+^ monocytes were then isolated from the mononuclear layer using magnetic bead separation with the Pan Monocyte Isolation Kit II (Miltenyi Biotech). CD14^+^ monocytes were plated in 24 well plates (Costar) at a concentration of 2 - 4 × 10^5^ cells/well in Iscove’s Modified Dulbecco medium (IMDM; Gibco) + 5 % autologous serum and 1% penicillin/streptomycin for 7 days at 37°C, 5% CO_2_ to allow them to mature into macrophages. Media was replaced after 3 days. In some instances, human MDMs were prepared by adherence of monocytes to tissue culture plates derived from the mononuclear layer of the Percoll^®^ gradients and washing off non-adherent lymphocytes (Rossi et al., 1998). Our previous studies have demonstrated that MDMs prepared by these protocols are active using a number of functional assays (efferocytosis, changes in receptor expression, mediator and cytokine release) (Rossi et al., 1998; Heasman et al., 2004).

Neutrophils were harvested from the granulocyte band and were routinely greater than 95% pure and over 99% viable. They were resuspended at 5 × 10^6^ cells/ml in IMDM +5 % autologous serum and 1% penicillin/streptomycin and cultured overnight at 37°C, 5% CO_2_ to allow them to become apoptotic. Levels of apoptosis were measured by morphological analysis of cytocentrifuge preparations and/or flow cytometry using Annexin V (Biolegend) and Draq7 staining (Biolegend).

For some studies, the human monocytic cell line, THP-1, was used. These cells were purchased from ATCC and cultured in Rosewell Park Memorial Institute (RPMI) 1,640 medium, 10% fetal calf serum (FCS), 1% penicillin/streptomycin and 1% l-glutamine (all Gibco) at 37°C, 5% CO_2._ The cells were maintained so that cell density did not exceed 1 × 10^6^ cells/ml.

CDK inhibitor drugs used were AT7519 (Astex Therapeutics, Astex, Cambridge, United Kingdom), R-roscovitine (Merck; 10–50 μM) or DRB (Merck; 10–50 μM). In all cases MDMs were pre-treated with specified concentrations of CDK inhibitors 1 h prior to stimulation with either 10 ng/ml, 100 ng/ml (siRNA experiments) *E. coli* derived LPS (O127:B8; Sigma Aldrich). For some studies, cells were also treated with 1 μM Dexamethasone (Sigma Aldrich).

### Cell Death Measurements

TUNEL staining was performed with the *In Situ* Cell Death Detection Kit, Fluorescein (Roche Applied Science, United Kingdom) according to the manufacturer’s instructions, 6 and 24 h after treatments. Cells were counter-stained with 1 μg/ml Hoechst 33342 (Life technologies). Images of the staining were taken using an EVOS™ fl auto2 fluorescence microscope (Thermo Fisher Scientific) and analysed using ImageJ software to calculate TUNEL positive cells as a percentage of the total population. Flow cytometry measurement of cell death was performed using Annexin V/Draq 7 (Biolegend) co-staining of cells, according to manufacturer’s instructions. Total percentage of double stained cells was used to quantify numbers of apoptotic cells in each condition.

### Efferocytosis Assay

Human MDMs were cultured as described above in 24 well plates for 7 days. On day 6 of the 7-days culture human neutrophils were isolated and induced to become apoptotic by aging overnight at a concentration of 5 × 10^6^ cells/ml in IMDM + 5% autologous serum and 1% penicillin/streptomycin. On day 7, apoptotic neutrophils were resuspended at 6 × 10^6^ cells/ml and stained with pHrodo green (Thermo Fisher Scientific) at a concentration of 1:10,000 for 30 min at room temperature. Prior to co-culture with pHrodo-stained apoptotic neutrophils, MDMs were treated with either AT7519 for 4 h or cytochalasin D (Sigma Aldrich) for 30 min at 37°C, 5% CO_2_ and then co-cultured at a ratio of 1 MDM:5 apoptotic neutrophils for 1 h at 37°C, 5% CO_2_. The MDMs were then removed from the plates with 0.05% Trypsin/EDTA (Thermo Fisher Scientific) and the levels of pHrodo^+^ efferocytosis were determined using a BD LSR four laser analyser flow cytometer (BD Biosciences). Data were analysed using Flowjo version 10.5.3 (TreeStar).

CDK9 siRNA-transfected MDMs were transfected at day 5 and left for a further 3 days. On day 8, 20 h aged apoptotic neutrophils were labelled with pHrodo as above. Transfected MDMs were then co-cultured with apoptotic neutrophils at a ratio of 1:20 (2 × 10^5^ MDMs: 4 × 10^6^ neutrophils) and left for 1 h at 37°C, 5% CO_2_ and the cells then lifted for flow cytometry as described above. Efferocytosis was then defined by pHrodo^+^ cell events analysed using the same cytometer and software described above.

### Quantitative Real Time PCR

To determine the expression of genes associated with macrophage polarisation and an inflammatory phenotype, RNA was extracted from MDMs pre-treated with AT7519 for 1 h followed by 10 ng/ml LPS for 6 and 24 h, using the RNAeasy mini kit (Qiagen) according to manufacturer’s instructions. RNA was then converted to cDNA using the Quantitect^®^ reverse transcription kit from Qiagen. Quantitative RT-PCR was performed on a StepOne real time PCR machine (Applied Biosystems) using PowerUp™ SYBR^®^ Green Master mix (Applied Biosystems) with predesigned KiCqStart SYBR^®^ Green primers from Sigma Aldrich, and analysed using the 2^−ΔCT^ method, compared to β-actin as a housekeeping gene. Primer sequences are listed in Table 1.

**TABLE 1:** KiCqStart SYBR^®^ Green primer sequences.

Gene	Forward Primer Sequence	Reverse Primer Sequence
CD80	AGG​AGG​AAT​GAG​AGA​TTG​AG	GAC​CTT​CAG​ATC​TTT​TCA​GC
HLA-DR	ATT​ATT​GGG​ACC​ATC​TTC​ATC	TAA​GAA​ACA​CCA​TCA​CCT​CC
CD206	AAA​TTT​GAG​GGC​AGT​GAA​AG	GGA​TTT​GGA​GTT​TAT​CTG​GTA​G
CD163	ATG​AGT​CCC​ATC​TTT​CAC​TC	CTA​TGT​CCC​AGT​GAG​AGT​TAC
IL-6	GCA​GAA​AAA​GGC​AAA​GAA​TC	CTACATTTGCCGAAGAGC
TNF	AGG​CAG​TCA​GAT​CAT​CTT​C	TTATCTCTCAGCTCCACG
IL-10	GCC​TTT​AAT​AAG​CTC​CAA​GAG	ATC​TTC​ATT​GTC​ATG​TAG​GC
IL-1β	CTA​AAC​AGA​TGA​AGT​GCT​CC	GGTCATTCTCCTGGAAGG
β-Actin	GAC​GAC​ATG​GAG​AAA​ATC​TG	ATG​ATC​TGG​GTC​ATC​TTC​TC

### Enzyme-Linked Immunosorbent Assay

ELISAs were used to determine the effects of CDKi on MDM inflammatory cytokine secretion. On day 7 of culture MDMs were pre-treated with concentrations of CDKi as indicated in the text for 1 h before treatment with 10 ng/ml LPS. At both 6 and 24 h of culture cell supernatants were removed and centrifuged at 300 g for 10 min to remove any cellular debris. Supernatants were stored at −20°C until analysis of IL-6, TNF and IL-10 secretion using DuoSet^®^ ELISA kits (R&D Systems). ELISAs were performed according to manufacturer’s instructions. Once developed, the optical density of each well was measured using a microplate reader at a wavelength of 450 nm. IL-1β secretion was measured using an IL-1β Human Uncoated ELISA kit (Life Technologies) according to the manufacturer’s instructions. IL-1β protein release was induced from human MDMs by stimulating with 10 ng/ml LPS for 3 h followed by an additional hour in the presence of 5 mM ATP (Sigma Aldrich).

### CDK9 siRNA Transfection

Prior to siRNA transfection, the media was changed on 5-days cultured MDMs to Opti-MEM reduced-serum medium (Gibco) and the cells left for approximately 1 h. MDMs were then transfected using Lipofectamine RNAiMAX (ThermoFisher Scientific) with either *SMARTpool: ON-TARGETplus human CDK9 siRNA* or *ON-TARGETplus Non-targeting Control Pool siRNA*, both 50 nM final concentration (Dharmacon, Horizon Discovery). MDMs were left to transfect for 3 days, after which they were stimulated for 24 h with 100 ng/ml LPS and supernatants were collected for cytokine analysis by ELISA. LDH was measured using a colorimetric assay kit (Roche, United Kingdom) and results were compared to a positive control (supernatants from MDM lysed using 2% Triton-X 100).

### Western Blotting

MDMs used for CDK9 siRNA transfection were lysed from duplicate wells (per treatment) with 100 µl of RIPA buffer (Sigma Aldrich) containing a 1:100 dilution of protease inhibitor cocktail (Sigma Aldrich). Cell lysates were subjected to three cycles of snap-freezing on dry ice and thawing on ice with 15 s of vortexing in-between each cycle. They were centrifuged at 12,000 *g* for 15 min at 4°C and the cell-free supernatants stored at −80°C until required. The protein concentration of cell lysates was determined *via* BCA Protein Assay Kit (Thermo Fisher Scientific). Western blotting was then carried out with approximately 15 µg of protein lysates run on 10-well NuPAGE 4–12% Bis-Tris Gels using an XCell II Blot Module System (both Thermo Fisher Scientific) according to manufacturer’s instructions. Briefly, lysates were transferred onto a polyvinylidene difluoride Immobilon-P Transfer Membrane (Millipore Sigma, Immobilon), blocked for 1 h at room temperature with 5% wt/vol dried milk made up in 1X Tris-buffered saline/0.1% Tween-20 before overnight incubation at 4°C (or 30 min at room temperature for *β*-actin) with primary antibody for CDK9 (1:1,000) or *β*-actin (1:50,000). Blots were then incubated with appropriate horseradish peroxidase-conjugated secondary antibody for either 1 h (CDK9, 1:2,500), or 30 min (*β*-actin, 1:10,000) at room temperature before incubation with ECL-prime (GE Healthcare), and exposed to double-sided light-sensitive X-ray film (Scientific laboratory Supplies) and processed through an X-ray developer Ecomax processor (Photon Imaging Systems). For CDK9, both 42 and 55 kDa isoforms were probed for using a CDK9 (C12F7) rabbit monoclonal primary antibody (Cell Signaling) and polyclonal goat anti-rabbit secondary antibody (Dako). To probe for *β*-actin, a mouse monoclonal anti-β-actin primary antibody (Sigma) and polyclonal goat anti-mouse secondary antibody (Dako) were used. Densitometry analysis was performed using ImageJ software (National Institutes of Health). Digital blots were converted to greyscale and for CDK9 siRNA transfected MDMs, the relative densities of both CDK9 isoforms (combined) and *β*-actin were calculated and then divided by each other respectively to determine the extent of CDK9 knockdown compared to control (untreated) MDMs.

### CDK9 Over-Expression in THP-1 Cells

CDK9 was overexpressed in THP-1 cells using a pcDNA3-CDK9 expression plasmid (Fujinaga et al., 1998), compared to mock-transfected cells and cells transfected with pMAX-GFP (Lonza) as a vector control (with GFP expression providing a measure of plasmid transfection efficiency). Pre-matured THP-1 cells were transfected by Amaxa Monocyte Nucleofector kit (Lonza), as per the following protocol adapted from Schnoor et al. (Schnoor et al., 2009). Briefly, 1.5 × 10^7^ THP-1 cells were plated in RPMI +100 ng/ml PMA in a 75 cm^2^ flask for 48 h. Cells were then detached with 5 ml of Accutase (Sigma) for approximately 30 min at 37°C. For each transfection, 2.5 × 10^6^ cells were transferred to a 1.5 ml Eppendorf in 100 µl Amaxa Nucleofector solution. 1 µg relevant plasmid DNA was added to each tube, and the solution transferred to cuvette. Cells were transfected with Amaxa Nucleofector program Y-001, immediately resuspended in 0.5 ml RPMI + 20% FCS, and transferred to a 6-well plate (Costar) containing an additional 1.5 ml RPMI + 20% FCS. Cells were allowed to recover for 4 h, then the media was replaced with fresh RPMI + 20% FCS and cells were stimulated ± 1 μg/ml LPS for 6 h. At 6 h, supernatants were removed to test cytokine levels by ELISA. The remaining cells were harvested for RNA using the RNAeasy kit (Qiagen), converted to cDNA with Quantitect^®^ RT kit from Qiagen and tested for expression of CDK9 by Real-Time PCR using a TaqMan primer-probe for CDK9 (Hs00977896_g1; Life Technologies) and *β*-actin (Hs01060665_g1; Life Technologies) as a house-keeping gene. CDK9 expression was assessed using the 2^−ΔCT^ method.

### Statistical Analysis

For TUNEL staining, efferocytosis, cytokine ELISA data, siRNA and CDK9 overexpression data intergroup significance was determined by a one-way ANOVA with a Tukey post-test. Statistical analysis was performed using GraphPad Prism 8 (GraphPad Software Inc., La Jolla, United States). A value of *p* ≤ 0.05 was considered significant.

## Results

### Pharmacological Inhibition of CDK9 Attenuates LPS-Induced Inflammatory Cytokine Release From Human MDMs

**FIGURE 1:**
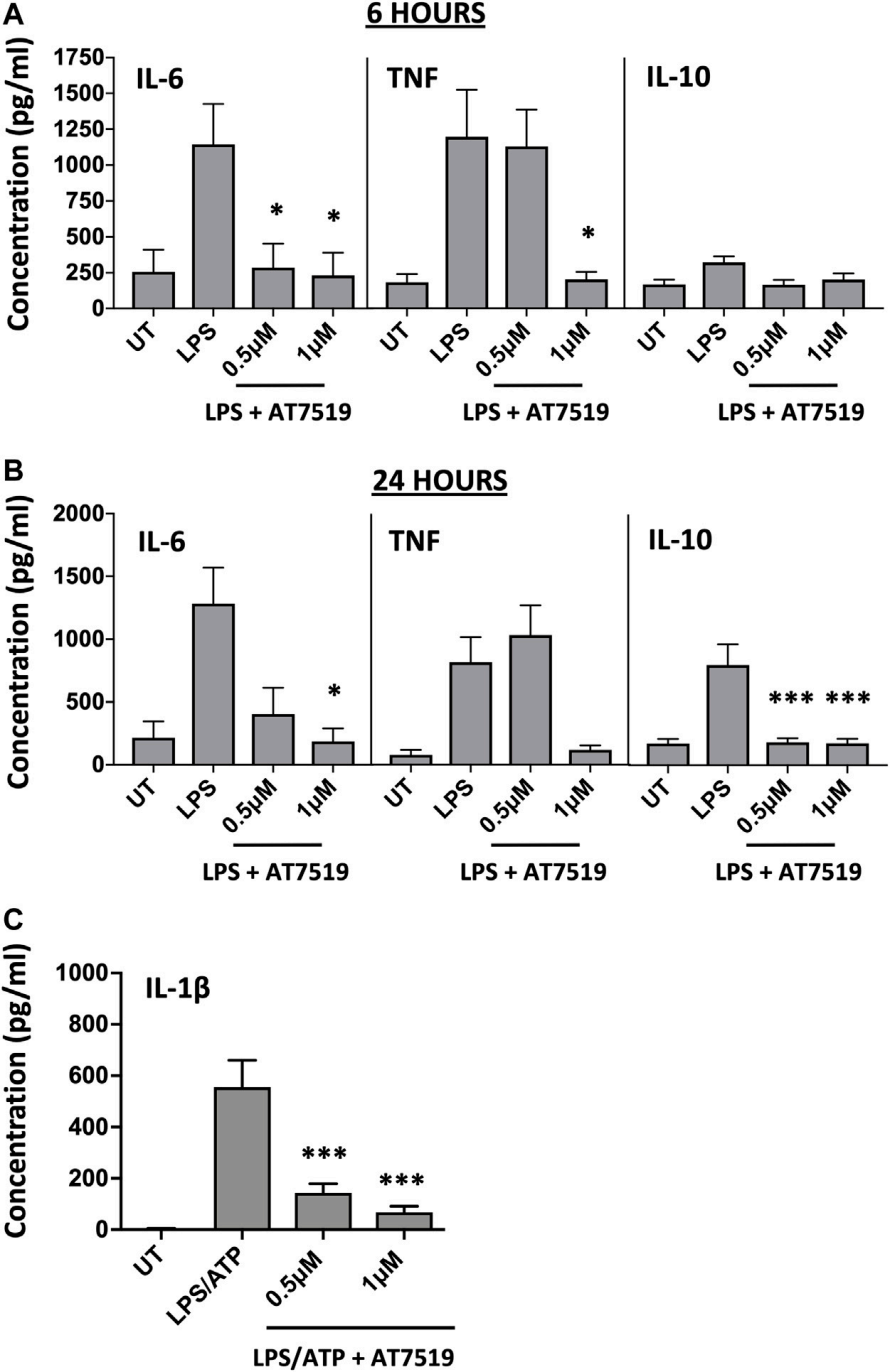
Pharmacological inhibition of CDK9 with AT7519 attenuates LPS-induced pro-inflammatory cytokine release from human MDMs. Panels **(A)** and **(B)** IL-6, TNF and IL-10 cytokine release by MDMs untreated (UT) with AT7519 with or without LPS at either 6 h **(A)** or 24 h **(B)**. Data represent means ± SEM from n = 6 separate donors, *p < 0.05 **p < 0.01 ***p < 0.001. **(C)** IL-1β protein expression by MDMs pre-treated with AT7519 with or without 10 ng/ml LPS (3 h) and 5 mM ATP (1 h). Data represent means ± SEM from *n* = 3 separate donors, *p < 0.05 **p < 0.01 ***p < 0.001.

Given that previous studies with AT7519 had shown its effects on the resolution of inflammation *in vivo*, we were interested to see what effect this CDKi had on inflammatory cytokine release and gene expression by LPS-stimulated MDMs. LPS was used to induce an acute pro-inflammatory phenotype in our MDMs. Treatment with 1 μM AT7519 was able to significantly decrease IL-6 and TNF protein release by LPS-stimulated MDMs at 6 h (Figure 1A) and 24 h (Figure 1B) and also reduced IL-10 protein release at the later timepoint of 24 h. Analysis of corresponding gene expression levels confirmed this effect on IL-6, TNF, and IL-10 expression following AT7519 and LPS treatment at 6 and 24 h (Supplementary Figures S1A, S1B). Similar effects on IL-6, TNF and IL-10 cytokine release were also observed using other pharmacological inhibitors of CDK9 such as R-roscovitine and DRB, but at higher concentrations than used for AT7519, starting at 10 µM (Supplementary Figures S2, S3).

We also looked at the effects AT7519 treatment had on MDM IL-1β release, another important cytokine in inflammatory disease. Treatment of MDMs with AT7519 was also able to inhibit the release of IL-β protein following LPS and ATP stimulation at 3 h (Figure 1C). Additionally, we demonstrated that gene expression levels of IL-1β induced in response to LPS were significantly inhibited at both 6 and 24 h respectively (Supplementary Figures S1C, S1D). Overall, these data suggest that AT7519 treatment inhibited LPS-induced pro-inflammatory cytokine secretion by macrophages.

### Pharmacological Inhibition of CDK9 by AT7519 Alters Markers of Polarisation

**FIGURE 2:**
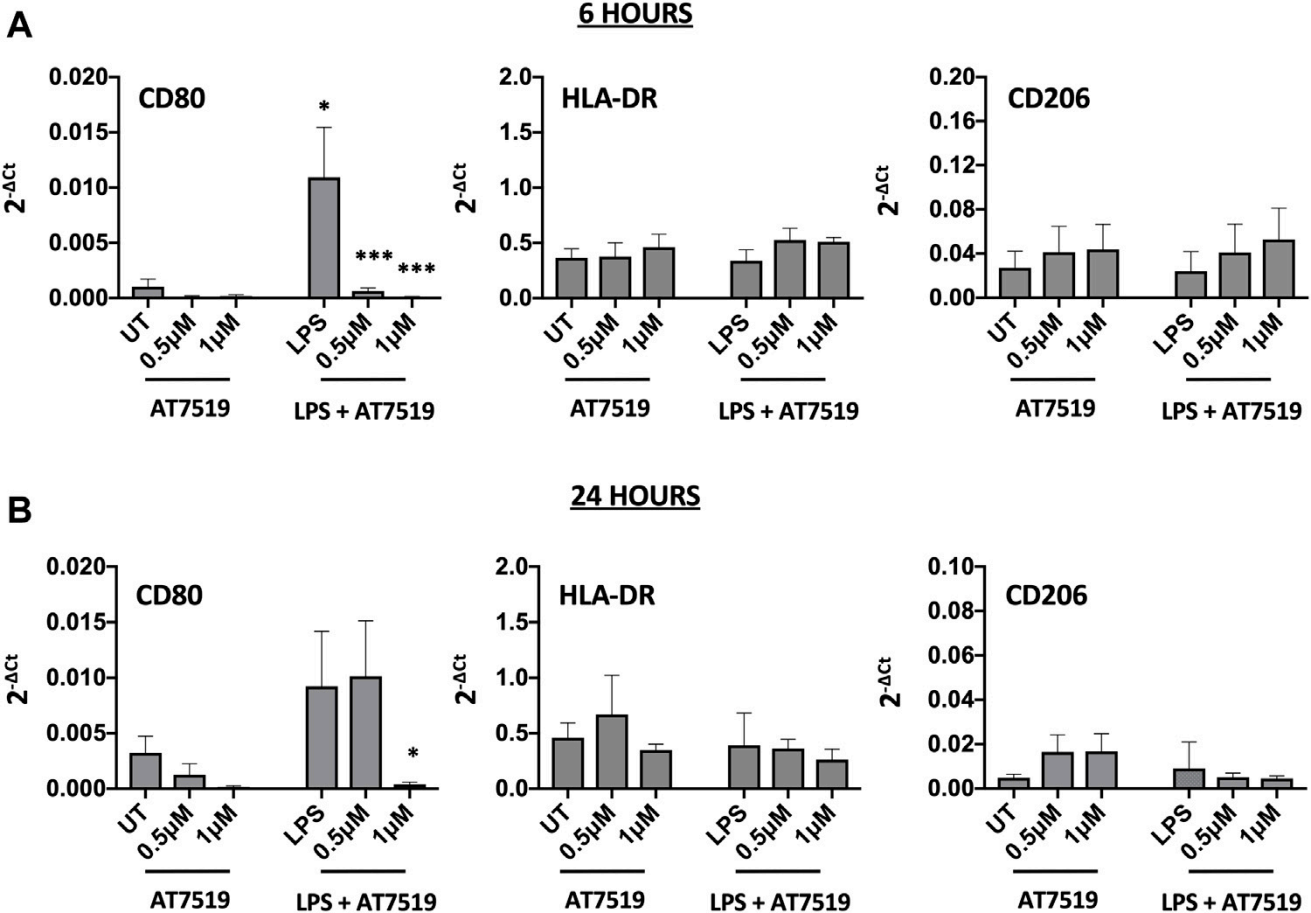
Pharmacological inhibition of CDK9 by AT7519 alters markers of pro-inflammatory polarisation. CD80, HLA-DR and CD206 mRNA expression levels by MDMs either untreated (UT) or pre-treated with AT7519 with or without LPS at either 6 **(A)** or 24 **(B)** hours. Data represent means ± SEM from *n* = 6 separate donors, *p < 0.05 **p < 0.01 ***p < 0.001.

MDMs cultured in the presence of LPS upregulate pro-inflammatory functions including CD80 and HLA-DR (Mills et al., 2000). To determine the effects of AT7519 on these markers we treated our cells with AT7519 and analysed gene expression of key markers. AT7519 was able to significantly downregulate LPS-mediated expression of the co-stimulatory molecule CD80 at both 6 (Figure 2A) and 24 (Figure 2B) hours in a concentration dependent manner. AT7519 had no effect on HLA-DR or on CD206, a marker of alternative activation, at either time point. Further, inhibition of CDK9 with AT7519 in the absence of LPS did not alter markers of LPS-stimulated polarisation at either timepoint. Together these data suggest that AT7519 down-regulates markers of inflammatory LPS-stimulated macrophages, highlighting a role of CDK9 in driving an inflammatory phenotype in human MDMs.

### Treatment of Human MDMs With AT7519 Does Not Affect Cell Viability

**FIGURE 3:**
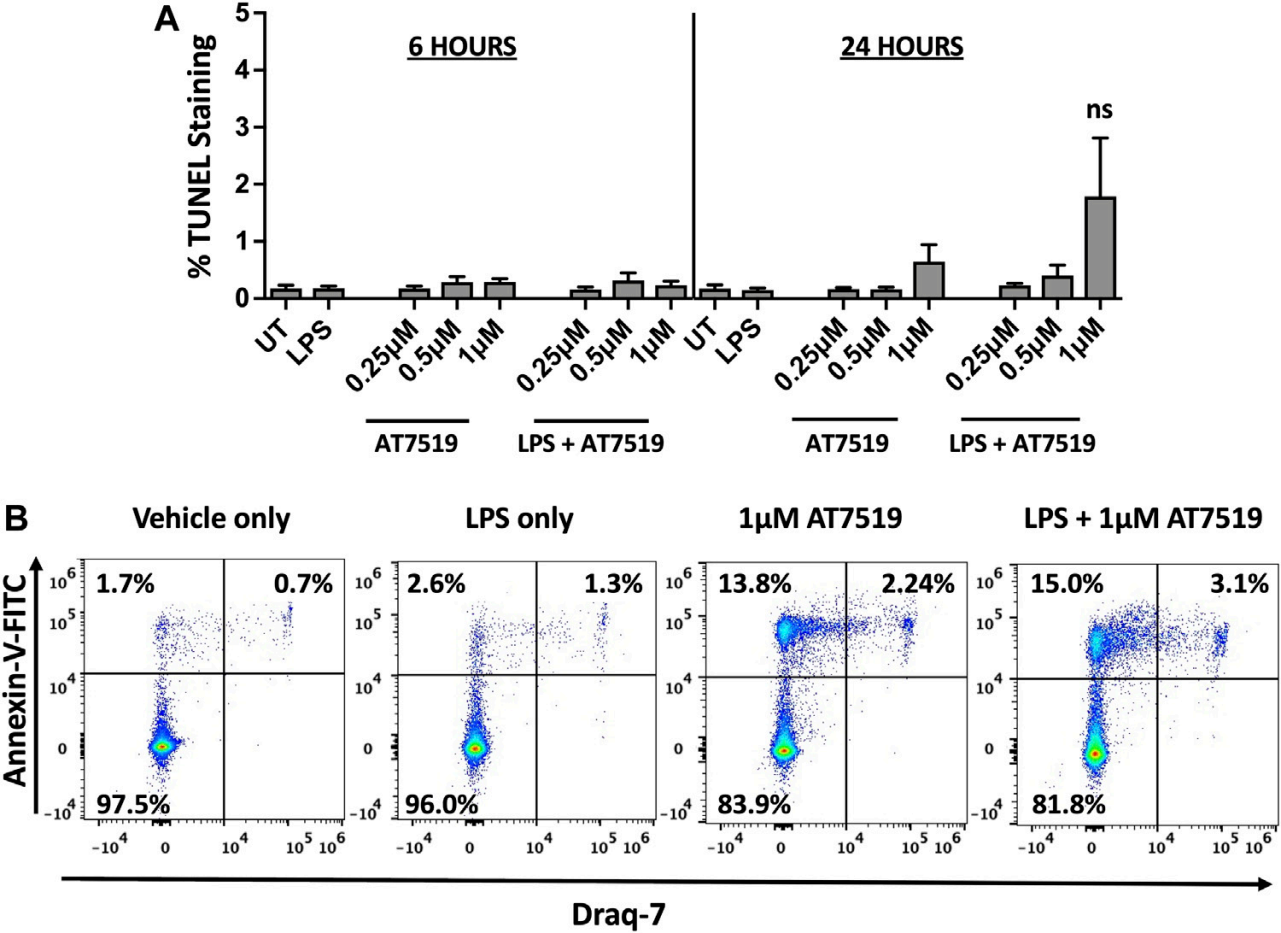
Treatment of human MDMs with AT7519 does not affect cell viability. **(A)** Quantitation of TUNEL positive MDMs treated ± AT7519 and ± LPS as shown, at 6 and 24 h. Quantitation is shown as the percentage of TUNEL positive cells compared to the total cell population stained with Hoechst. UT = untreated. ns = no significant difference. **(B)** Representative Annexin V/Draq-7 flow cytometry plots of MDMs treated with vehicle-only, LPS-only, 1μM AT7519, or 1 μM AT7519 + LPS, showing no increase in Annexin V/Draq-7 double positive cell numbers (upper right quadrants) in cells incubated with AT7519 + LPS versus AT7519 alone.

We previously reported that treatment with CDK9 inhibitors such as R-roscovitine, DRB and AT7519 induce apoptosis of human neutrophils (Leitch et al., 2012; Lucas et al., 2014), so we investigated whether AT7519 had a similar effect on MDMs. MDMs were treated with increasing concentrations of AT7519, including concentrations known to induce neutrophil apoptosis (1 μM), with or without LPS, and levels of cell death was assayed using TUNEL staining at 6 and 24 h. We observed that AT7519, either alone or in the presence of LPS, does not induce significant levels of cell death (Figure 3A). To further differentiate levels of cell death in these conditions, we stained cells treated with vehicle-only, LPS-only, 1 μM AT7519, or 1 μM AT7519 + LPS with Annexin V and Draq7 and examined them by flow cytometry. We observed no increase in numbers of MDMs that stained positively for Annexin V and Draq7 when incubated with 1 µM AT7519 ± LPS, indicating no increase in MDM cell death at levels of AT7519 that were previously associated with induction of apoptosis in neutrophils. (Figure 3B).

### Pharmacological Inhibition of MDMs With AT7519 Does Not Affect Macrophage Initial Efferocytosis

**FIGURE 4:**
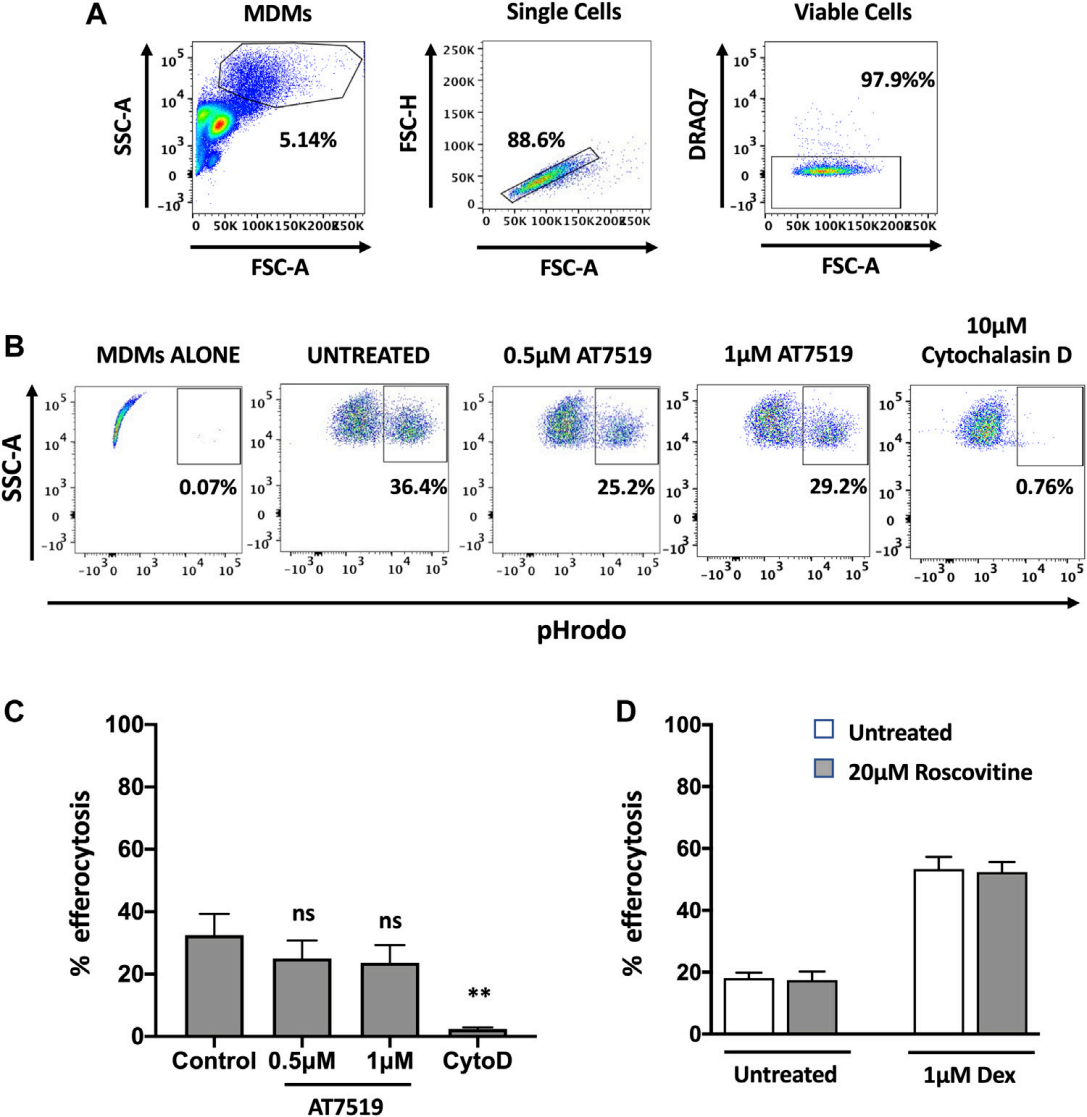
Pharmacological inhibition of MDMs with AT7519 does not affect macrophage initial efferocytosis. **(A)** Gating strategy used for the analysis of MDMs, analysing viable single cells from the MDM population. **(B)** Representative flow cytometry plots depicting the effects of AT7519 pre-treatment on macrophage efferocytosis of pHrodo labelled apoptotic neutrophils at a 1 h timepoint. Macrophages that had undergone efferocytosis are shown as pHrodo positive. **(C)** Collated levels of efferocytosis at 1 h timepoint from all donors tested, comparing control treated MDMs to MDMs treated with 0.5 µM or 1µM AT7519, or 10 µM Cytochalasin D (Cyto D). **(D)** Quantitation of efferocytosis at 1 h timepoint of apoptotic neutrophils in MDMs treated ± 20 µM Roscovitine, +/− 1 µM Dexamethasone. Data represent means ± SEM from *n* = 6 separate donors, **p < 0.01. ns = no significant difference.

Having previously shown that AT7519 can promote the resolution of neutrophilic inflammation through the induction of neutrophil apoptosis (Rossi et al., 2006), we next wanted to determine whether AT7519 can also promote inflammatory resolution by permitting treated MDMs to undergo efferocytosis of apoptotic neutrophils, a key step in the inflammatory resolution process. We therefore measured levels of efferocytosis at a single timepoint - MDMs were pre-treated with AT7519 for 4 h and co-cultured with pHrodo stained apoptotic neutrophils for 1 h before their initial levels of efferocytosis were determined using flow cytometry (Figures 4A,B). Baseline efferocytosis of apoptotic neutrophils by MDMs was approximately 32% and this was not significantly affected upon treatment with AT7519 (Figure 4C). To confirm efferocytosis could be blocked, cells were pre-treated for 30 min with cytochalasin D (a cell-permeable inhibitor of actin polymerization that blocks phagocytosis including efferocytosis (Lucas et al., 2014)), which almost completely inhibited MDMs’ ability to efferocytose apoptotic neutrophils. We also demonstrated that treatment with R-roscovitine also had no effect on the initial efferocytosis of pHrodo labelled apoptotic neutrophils by MDMs and that treatment with this CDKi had no effect on the up-regulation of efferocytosis by dexamethasone (Figure 4D), a glucocorticoid that augments efferocytosis (Liu et al., 1999). Overall, these data suggest that MDMs are able to efficiently phagocytose apoptotic neutrophils in the presence of CDK9 inhibitors AT7519 and R-roscovitine.

### Genetic Inhibition of CDK9 is Sufficient to Attenuate LPS-Induced Pro-Inflammatory Cytokine Release From Human MDMs

**FIGURE 5:**
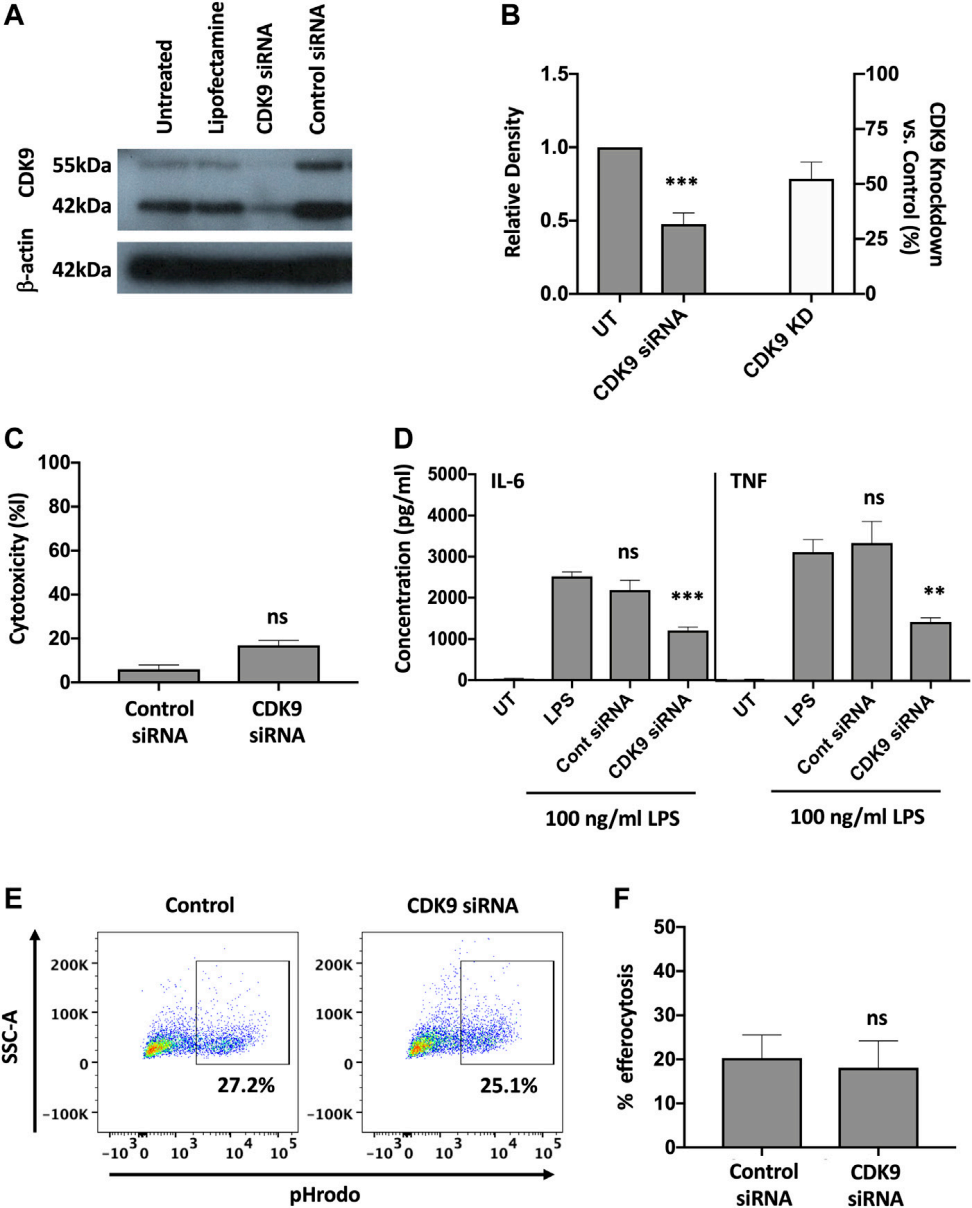
Genetic inhibition of CDK9 attenuates LPS-induced pro-inflammatory cytokine release from human MDMs. **(A)** Western blot of CDK9 protein expression in untreated (UT), lipofectamine-only treated, CDK9 siRNA and control siRNA treated MDMs, compared to Beta-actin as a loading control. Endogenous CDK9 is detected in MDMs as two isoforms of 42 and 55 kDa. **(B)** The relative expression density of CDK9 in UT versus CDK9 siRNA treated cells as measured by densitometry. **(C)** Cytotoxicity in control and CDK9 siRNA-treated MDMs as measured by LDH cytotoxicity assay. **(D)** IL-6 and TNF cytokine release from CDK9 siRNA treated MDMs stimulated with LPS for 24 h compared to controls. **(E)** Representative flow cytometry plots showing efferocytosis of pHrodo labelled apoptotic neutrophils in CDK9 siRNA-treated compared to control siRNA-treated MDMs. **(F)** Collated levels of efferocytosis from all donors tested, comparing control siRNA-treated and CDK9 siRNA-treated MDMs. Data represent means ± SEM from *n* = 6 separate donors, *p < 0.05 **p < 0.01 ***p < 0.001. ns = no significant difference.

To investigate whether inhibition of CDK9 was sufficient to attenuate cytokine release in human MDMs we genetically targeted CDK9 expression by siRNA knock-down. A significant reduction in CDK9 protein was achieved (Figures 5A,B). Transfection of MDMs with CDK9 siRNA did not significantly increase LDH levels compared to control siRNA transfection, as measured by an LDH cytotoxicity assay, suggesting siRNA knock-down of CDK9 had minimal cytotoxicity (Figure 5C). In agreement with our results using pharmacological inhibition in Figures 1, 4, siRNA knock-down of CDK9 expression in MDMs resulted in a significant downregulation in both IL-6 and TNF release from MDMs stimulated with LPS (Figure 5D), but did not affect the ability of these cells to undergo initial efferocytosis (Figures 5E,F). These data therefore indicate that CDK9 is responsible for driving LPS-induced proinflammatory cytokine release from human MDMs.

### Over-Expression of CDK9 Augments LPS-Induced Pro-Inflammatory Cytokine Release From THP-1 Cells

**FIGURE 6:**
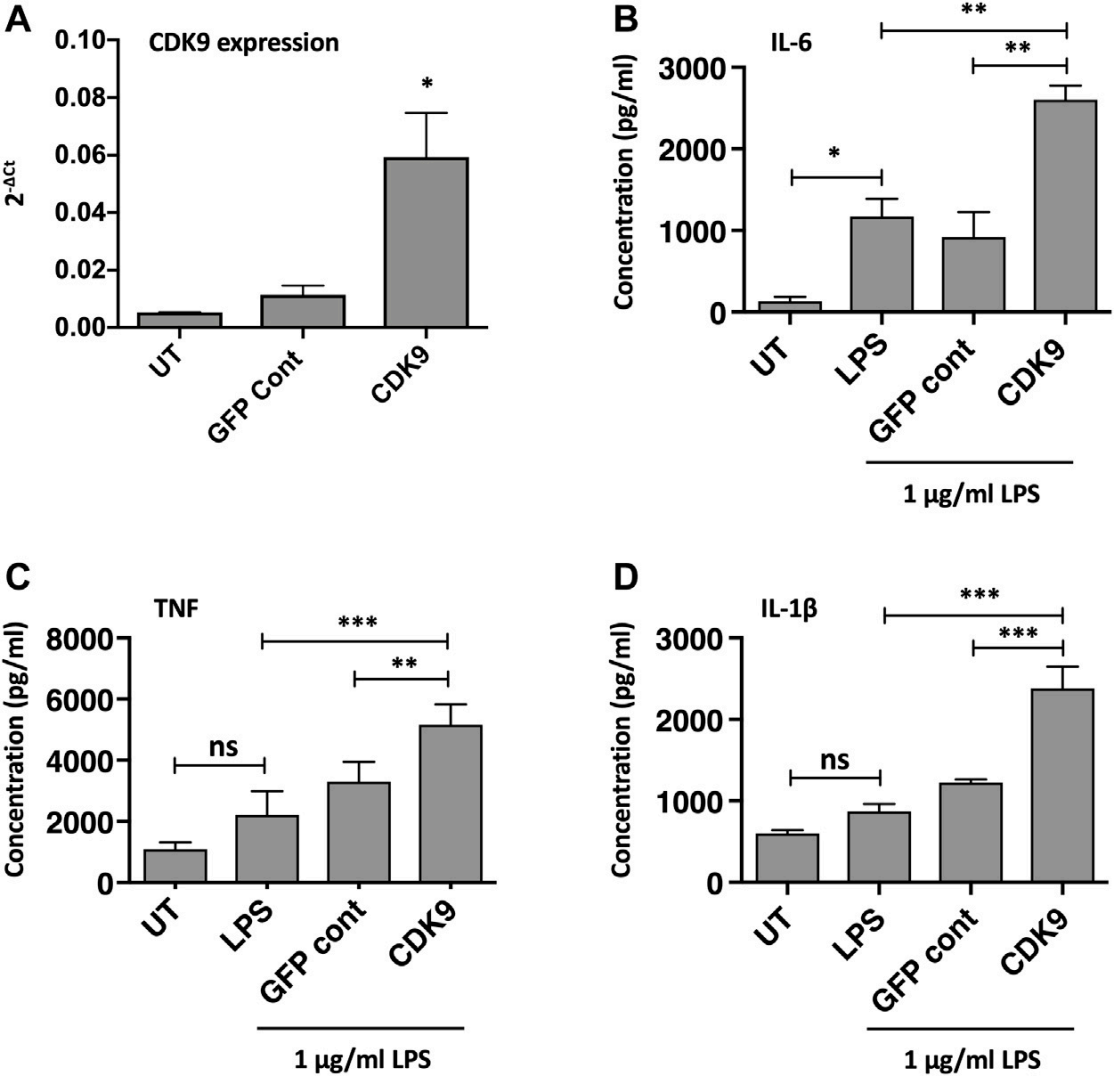
Genetic over-expression of CDK9 augments LPS-induced pro-inflammatory cytokine release from THP-1 cells. **(A)** CDK9 mRNA expression in THP-1 cells in either untreated (UT), GFP control and CDK9 plasmid transfected THP-1 cells, assayed by Real Time PCR. **(B–D)** Cytokine release determined by ELISA, comparing release of IL-6 **(B)**, TNF **(C)** and IL-1β **(D)** cytokines by untransfected (UT), GFP control and CDK9 plasmid transfected cells in response to 6 h stimulation with 1 μg/ml LPS. Data represent means ± SEM from *n* = 3 experiments, *p < 0.05 **p < 0.01 ***p < 0.001.

The role of CDK9 in driving inflammatory cytokine production was further supported by genetic overexpression of CDK9. As CDK9 plasmid transfection in MDMs did not achieve high-transfection efficiency in our hands, we therefore examined CDK9 overexpression in the monocyte-like cell line THP-1. Figure 6A shows a significant increase in CDK9 mRNA expression following CDK9 plasmid transfection of PMA-matured THP-1 cells compared to non-transfected and vector control transfected cells. This genetic overexpression of CDK9 mRNA in THP-1 cells resulted in a significant increase in LPS-mediated IL-6 (Figure 6B), TNF (Figure 6C) and IL-1β (Figure 6D) cytokine release. These data provide further support for the involvement of CDK9 in driving inflammatory cytokine secretion and suggest that inhibition of CDK9 may provide a novel way to attenuate an inflammatory phenotype in these cells.

## Discussion

Our previous data demonstrated that pharmacological inhibition of CDK9 induces neutrophil apoptosis and promotes the resolution of inflammation, making CDK9 a potential attractive therapeutic target to treat inflammatory diseases. Successful resolution of inflammation also requires that macrophages clear apoptotic neutrophils, ensuring they do not become necrotic and exacerbate tissue injury. Here we show that AT7519 can inhibit a proinflammatory phenotype in MDMs without affecting their ability to undergo efferocytosis (although it should be noted that we assessed initial efferocytosis at a single timepoint, rather than the kinetics of sustained efferocytosis). This is important, as failure of MDMs to undergo efferocytosis has been linked to several inflammatory lung diseases, autoimmunity and atherosclerosis (Szondy et al., 2014; Yurdagul et al., 2017; Kawano and Nagata, 2018). We also demonstrated that the CDKi R-roscovitine does not affect the upregulation of efferocytosis caused by the steroidal anti-inflammatory dexamethasone. Dexamethasone is often used as a therapeutic to treat various inflammatory conditions exemplified by its universal use in the treatment of hospitalised COVID-19 patients (Horby et al., 2021). Our data therefore suggest that CDKi drugs could be used successfully in conjunction with well-established steroid anti-inflammatory drugs such as dexamethasone. In this study we used LPS treatment to induce an acute pro-inflammatory state in our MDMs, rather than develop a chronic state of inflammation, and this is a potential limitation of the study. Future work will examine the effects of other stimuli such as LPS/IFNγ stimulation to induce a classical M1 phenotype.

During this study we demonstrated that CDK9 inhibitors AT7519 and R-roscovitine inhibit macrophage pro-inflammatory cytokine production, as well as their polarisation into a pro-inflammatory phenotype. These effects are significant in the context of inflammatory disease as it suggests that AT7519 may represent an important novel pharmacological agent to help treat inflammatory diseases. The ability of AT7519 to inhibit both IL-6 and TNF production is consistent with a role for CDK9, in complex with PTEFb, in the transcriptional regulation of inflammatory cytokines. Recent evidence has shown that pro-inflammatory macrophages can secrete large amounts of IL-10 (Jaguin et al., 2013; Tarique et al., 2015) however its inhibition by AT7519 is interesting since it is usually associated with anti-inflammatory responses (Voll et al., 1997). Our findings are in keeping with those of Zoja et al., who demonstrated that R-roscovitine (seliciclib) inhibited IL-10 release from concanavalin A activated splenocytes from control and NZB x NZW mice (Zoja et al., 2007). In this study the authors discuss the importance of IL-10 in driving B cell proliferation and survival highlighting an important inflammatory function for this cytokine and a potential therapeutic effect of AT7519. However, it is important to note that the full effects of AT7519 on other anti-inflammatory molecules and or cells should be investigated further to fully determine its effects on this important arm of the immune response. For example, we note in our study that AT7519 only has an effect on IL-10 at the later 24 h timepoint, whereas effects on IL-6 and TNF are observed at 6 h. The kinetics of IL-10 secretion by pro-inflammatory cells is clearly delayed in comparison to that of classical pro-inflammatory cytokines such as TNF and IL-6, whose secretion is stimulated by LPS at 6 h and remains relatively steady at 24 h. We plan to analyse these discrepancies in future studies, as well as examining IL-10 kinetics in cells where CDK9 has been genetically manipulated (e.g. by siRNA).

In line with its effects on pro-inflammatory cytokine secretion, AT7519 downregulated CD80 gene expression which is associated with an inflammatory phenotype in macrophages at both 6 and 24 h. This supports data showing that R-roscovitine significantly inhibited the activation marker iNOS mRNA and protein expression in LPS stimulated RAW264.7 cells (Du et al., 2009). Interestingly, AT7519 did not, however, induce expression of genes associated with an alternatively activated phenotype, exemplified by CD206 expression levels in Figures 2A,B, suggesting that it does not act to skew MDMs towards an anti-inflammatory state. Whilst we did not seek to actively induce classical and alternatively activated macrophage phenotypes prior to addition of AT7519, by stimulation with IFN*γ* + LPS or IL-4 and/or IL-13 respectively, we postulate that AT7519 is acting to suppress the acute pro-inflammatory phenotype induced by LPS alone in these cells. Further study of AT7519 in cells actively induced to classical/alternatively activated phenotypes would be beneficial. The interaction between CD80 and CD28 on T cells promotes T cell responses by decreasing apoptosis and increasing expansion of antigen specific T cells (Boise et al., 1995; Schweitzer and Sharpe, 1998). This interaction has also been shown to be important in promoting Th1 responses (Lenschow et al., 1996). This suggests that one of the ways AT7519 dampens inflammatory responses is by making MDMs less efficient at triggering antigen specific T cell responses. We plan to analyse other markers of macrophage polarisation in future studies (e.g. CD86 for pro-inflammatory and CD163 for alternatively activated macrophages), to help evaluate the effects of these drugs on macrophage differentiation state.

Interestingly, AT7519 did not significantly induce cell death in macrophages, whereas we showed previously that it strongly induced programmed cell death in neutrophils (Lucas et al., 2014). Our previous study showed that the AT7519 effect on neutrophil apoptosis was related to downregulation of Mcl-1 expression. Although we have also demonstrated that Mcl-1 expression is reduced in macrophages after CDKi treatment we believe that in macrophages other survival signals are important in these cells that prevent them from dying by apoptosis (Lucas et al., 2014).

**FIGURE 7:**
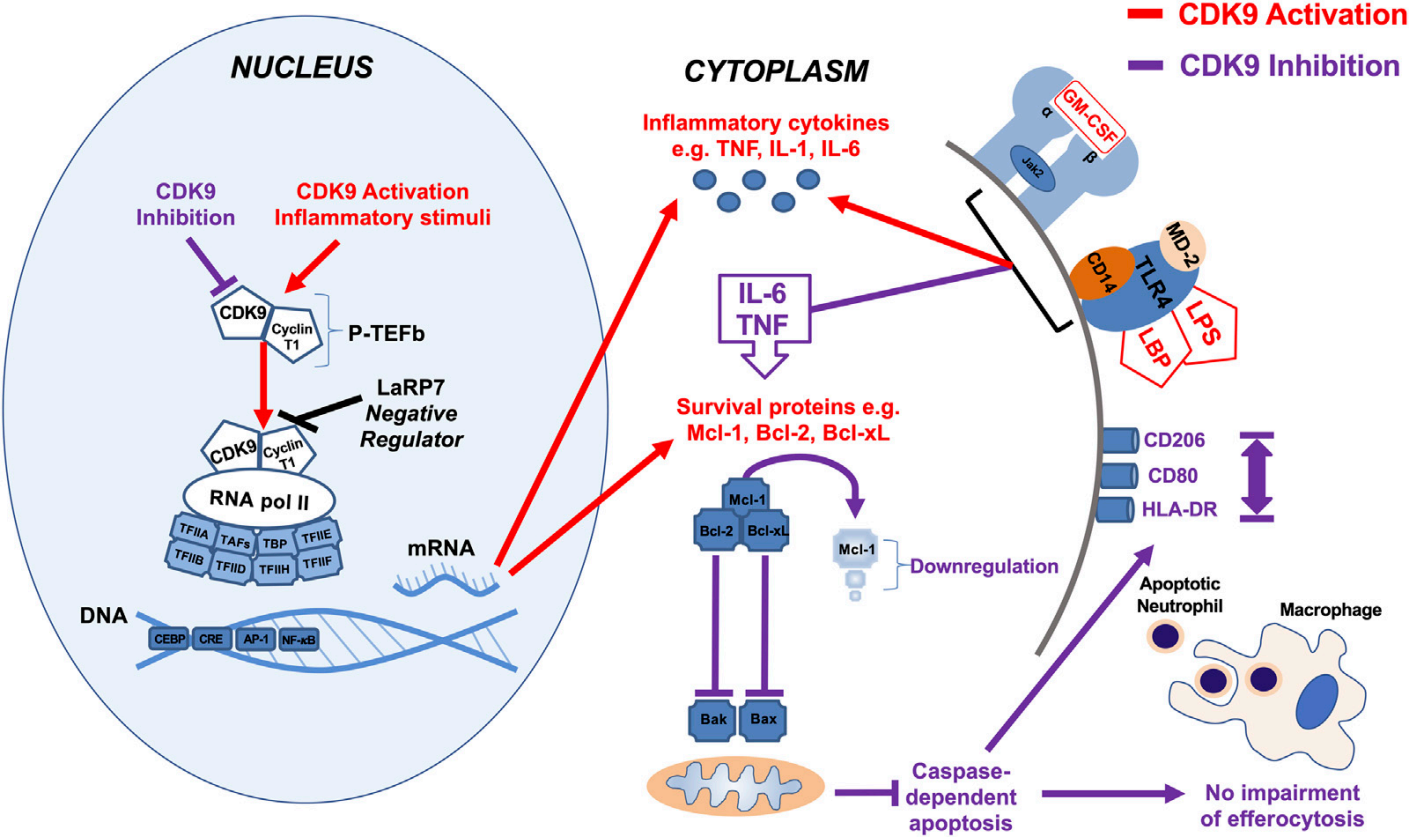
Summary diagram of effects of CDK9 inhibition and activation on inflammatory pathways in monocyte-derived macrophages. Activation of CDK9 initiates binding with regulatory subunit cyclin T to form the heterodimeric complex P-TEFb, which in turn regulates transcription elongation via phosphorylation of RNA pol II and subsequent mRNA synthesis of anti-apoptotic proteins and inflammatory cytokines. Inhibition of CDK9 in MDMs does not drive caspase-dependent apoptosis despite Mcl-1 downregulation, in contrast to its effects in neutrophils, as MDMs possess additional anti-apoptotic proteins such as Bcl-2 and Bcl-xL (Lucas et al., 2014). Inhibition of CDK9 can also reduce the release of potent inflammatory cytokines, including TNF, IL-6, and IL-1β after MDM stimulation with LPS, but does not impair MDM efferocytosis of apoptotic neutrophils, nor does it polarize MDMs to a pro- or anti-inflammatory phenotypes, based on cell surface markers.

We show that the effects of AT7519 are specific to CDK9 by genetically inhibiting it in human MDMs using siRNA. In doing so we were able to replicate the effects using AT7519 treatment on macrophages showing that siRNA knockdown of CDK9 significantly inhibited LPS induced IL-6 and TNF secretion but had no effect on the ability of MDMs to undergo efferocytosis. This suggests the effects we observe with AT7519 are specific to CDK9. To further support this we then overexpressed CDK9 in THP-1 cells using CDK9 plasmid transfection. This resulted in increased mRNA expression in the cells as well as increased IL-6, TNF and IL-1β release compared to LPS alone, suggesting that CDK9 plays a role in the transcription of these proinflammatory molecules and provides a pathway by which its inhibition can affect MDM phenotype (Figure 7).

Interestingly, in support of our data it has been demonstrated that the endogenous natural CDK inhibitor p21 (waf1/CIP1), acts as a negative feedback system that controls macrophage activation (Lloberas and Celada, 2009). The study by Scatizzi et al., showed that the CDK domain of p21 is a suppressor of IL-1β-mediated inflammation in activated macrophages (Scatizzi et al., 2009). They reported that mice deficient in p21 (p21 (−/−)) displayed increased susceptibility to endotoxic shock, which is associated with increased serum levels of IL-1β (from macrophages). Interestingly, they also reported that siRNA knockdown of p21 in human macrophages resulted in increased IL-1β secretion. In the same journal issue Trakala et al., show that p21(WAF1/CIP1) regulates macrophage activation and septic shock susceptibility linking their findings to p21 mediated control of NF-κB-associated inflammation (Trakala et al., 2009). Our results, where we pharmacologically and genetically inhibited CDK9 are consistent with these findings. Gerlach et al. have recently shown that nucleotides from efferocytosed apoptotic cells can trigger macrophage proliferation to improve resolution of inflammation (Gerlach et al., 2021). Interestingly, Arpa et al. demonstrated that the type 2-type cytokine IL-4 blocks M-CSF-dependent macrophage proliferation by inducing p21Waf1 in a STAT6-dependent way suggesting that macrophage proliferation could be regulated by CDKi drugs (Arpa et al., 2009). To our knowledge, AT7519 does not have any direct effect on endogenous CDK inhibitors such as p21 (waf1/CIP1), but it would be of interest to investigate how AT7519 may modulate efferocytosis-induced macrophage proliferation. In combination with further studies on continual/late efferocytosis (as opposed to our initial efferocytosis observations reported here), this would give an important insight into how CDKi drugs would impact ongoing or chronic inflammatory disease.

Overall, we have demonstrated that AT7519 targets key inflammatory pathways in MDMs to inhibit inflammatory cytokine secretion and the upregulation of inflammatory genes (Figure 7). Importantly however, these CDKi’s do not affect the ability of MDMs to undergo efferocytosis of apoptotic neutrophils, a critical step in inflammatory resolution. As a result, our data suggest the CDKi’s such as AT7519 and R-roscovitine represent important pharmacological agents which could be used in the treatment of inflammatory disease (e.g., ARDS and COVID-19) either alone or in combination with glucocorticoid anti-inflammatory drugs such as dexamethasone.

## Data Availability

The raw data supporting the conclusion of this article will be made available by the authors, without undue reservation.
