# Supplementary material for: Inhibition of Cyclin-Dependent Kinase 9 Downregulates Cytokine Production Without Detrimentally Affecting Human Monocyte-Derived Macrophage Viability
Source: Front Cell Dev Biol. 2022 May 26;10:905315. doi: 10.3389/fcell.2022.905315 (PMC9178253; doi:10.3389/fcell.2022.905315)
Supplement: Supplementary file 2 [file DataSheet3.pdf]

Supplementary Figure S3.

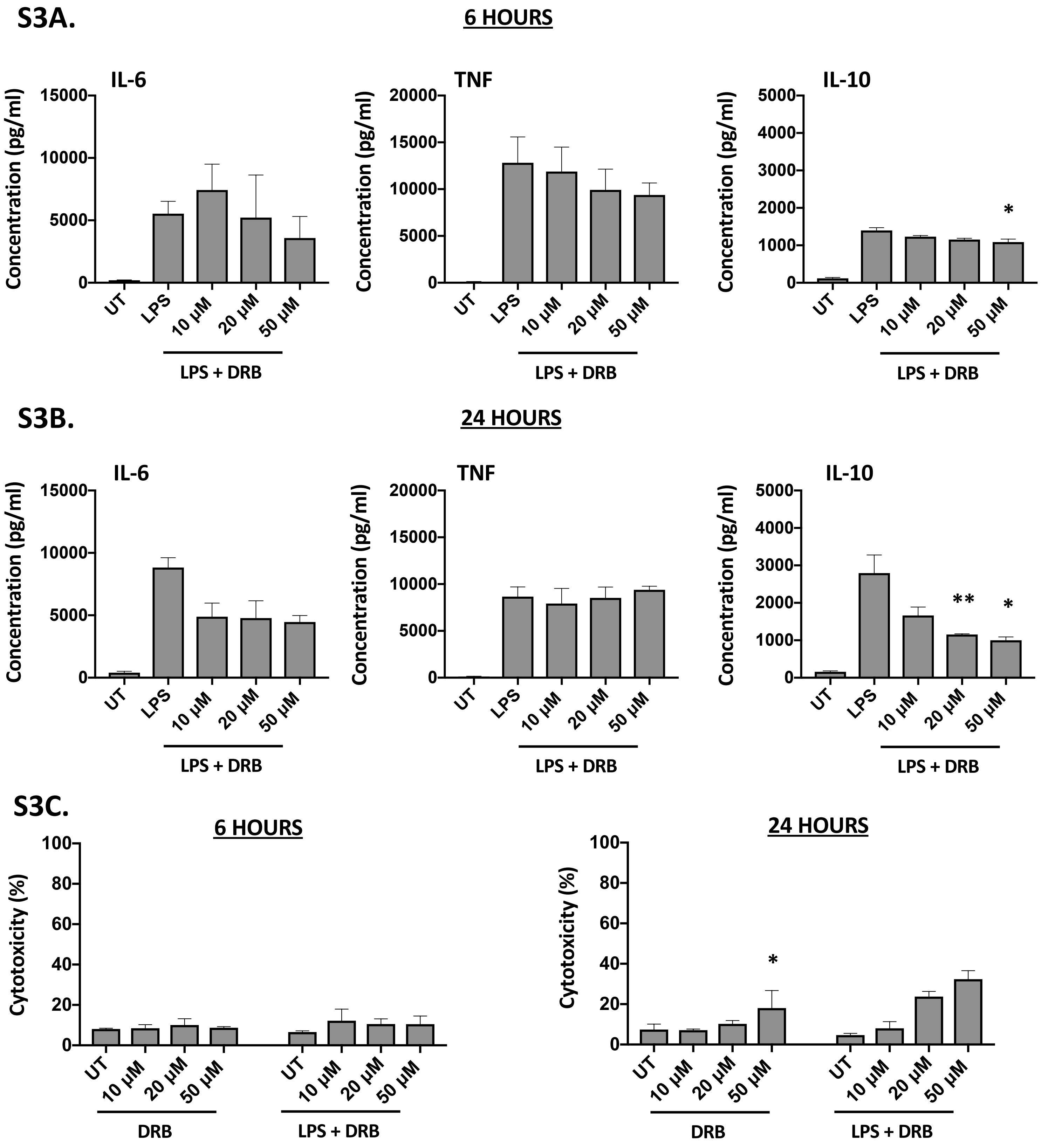

Supplementary Figure S3: Pharmacological inhibition of CDK9 with DRB attenuates LPS-induced pro-inflammatory cytokine release from human MDMs. IL-6, TNF and IL-10 protein expression by MDMs pre-treated with increasing concentrations of R-DRB with or without LPS at either 6 (S3A) or 24 (S3B) hours. S3C) Levels of cytotoxicity in MDMs pre-treated with increasing concentrations of R-DRB with or without LPS at either 6 or 24 hours, as assayed by LDH release. n=2-6 *P<0.05 **P<0.01 ***p<0.001.
