# Supplementary material for: Inhibition of Cyclin-Dependent Kinase 9 Downregulates Cytokine Production Without Detrimentally Affecting Human Monocyte-Derived Macrophage Viability
Source: Front Cell Dev Biol. 2022 May 26;10:905315. doi: 10.3389/fcell.2022.905315 (PMC9178253; doi:10.3389/fcell.2022.905315)
Supplement: Supplementary file 3 [file DataSheet1.pdf]

Supplementary Figure S1.

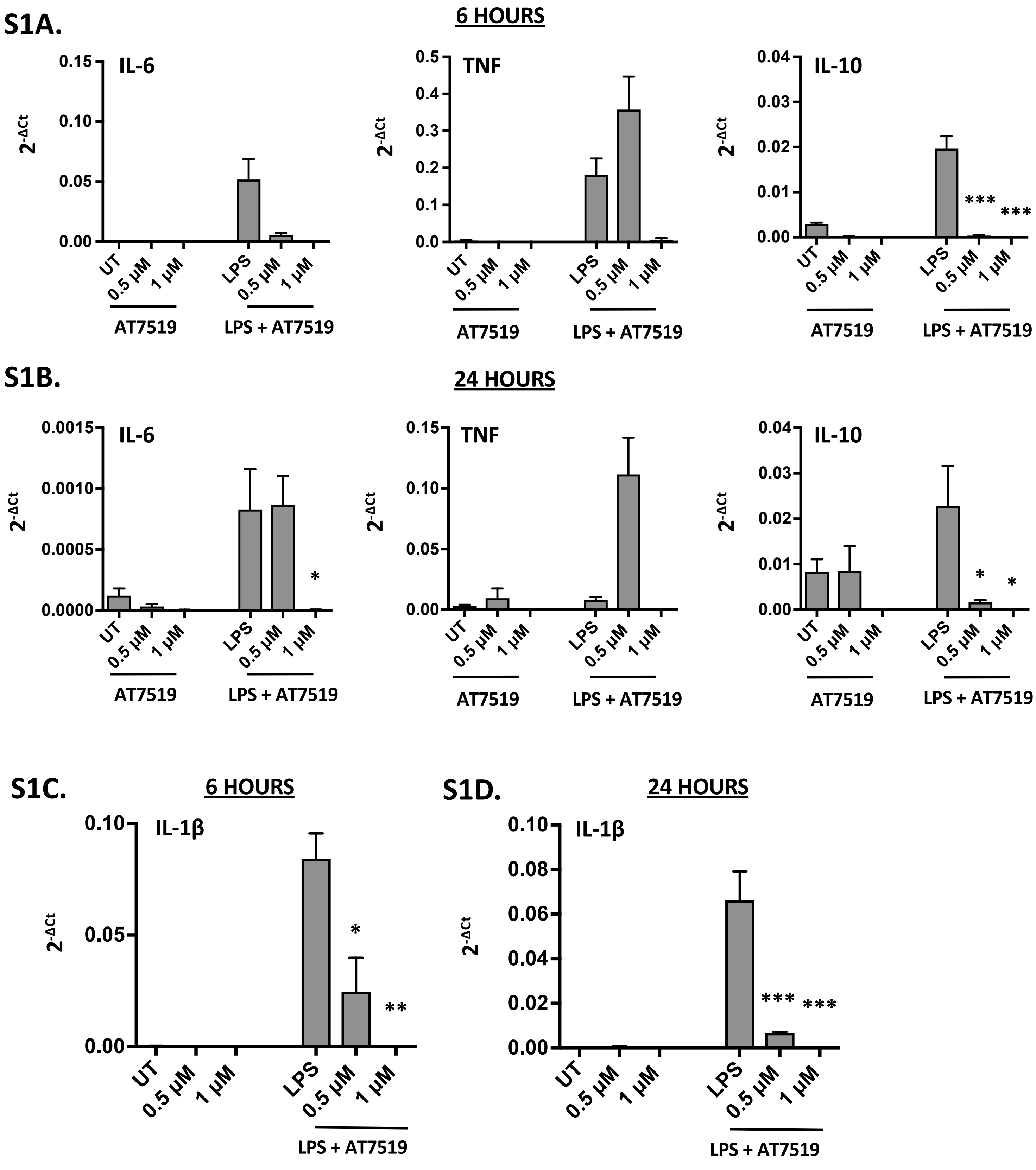

Supplementary Figure S1: Pharmacological inhibition of CDK9 with AT7519 reduces transcription of proinflammatory cytokines. Panels S1A and S1B: IL-6, TNF and IL-10 mRNA expression levels by MDMs pre-treated with AT7519 with or without LPS at either 6 hours (S1A) or 24 hours (S1B). Panels S1C and S1D: IL-1β mRNA expression levels by MDMs pre-treated with AT7519 with or without LPS at either 6 hours (S1C) or 24 hours (S1D). n=3 *P<0.05 **P<0.01 ***p<0.001.
